# Observations and Experiments on Softening, Erosion, and Perforation of the Stomach

**Published:** 1837

**Authors:** Henry Imlach


### II.—ANALYTICAL.

**Art. I.—Observations and Experiments on Softening, Erosion, and Perforation of the Stomach. By Henry Imlach, M. D.**

In a late number of the Edinburgh Medical and Surgical Journal, Dr. Imlach has presented the profession with a highly valuable paper on the above lesions, the most interesting portions of which we shall extract for the benefit of our readers, as the entire essay is too long for our Analytical department. It is well known that this interesting subject was first introduced to the notice of medical men by the great John Hunter, in a paper which he read to the Royal Society of London, in 1772. He supposed that the lesions in question resulted altogether from the action of the gastric juice upon the coats of the stomach after death, and he found, in several instances, that the influence of this fluid extended to some of the surrounding organs, such as the spleen, liver, diaphragm, and even the lungs. This theory of the proximate cause of softening, erosion, and perforation of the stomach has since been disputed by other writers, especially by the French and German, though, it is believed, by no means successfully. The paper of Dr. Imlach embraces not only an account of his own observations and experiments, the latter of which he performed conjointly with his friend Dr. Simpson, but a full analysis of the opinions of the principal writers on the subject, from the time of Mr. Hunter until the present moment. The author thus describes the anatomical characters of these singular lesions, and, as will be seen in a subsequent paragraph, his conclusions as to their proximate cause, fully coincide with those of John Hunter, Prof. Carswell, and other pathologists.

"In the first stage of softening, the villous coat is found thinner and paler than in the healthy state. Neither around the softening, nor underneath it, nor in the affected part itself, nor even in the portion of the mesentery corresponding to it, are there found any injected vessels, to indicate pre-existing inflammation. When viewed through a magnifying glass, the villous membrane appears flaccid, its villous prominences are collapsed, pale, whitish, and yet seem to retain their proper texture, but on being rubbed in the slightest manner, even by the point of the finger, it is converted into a thick mucous-like pap. If in the softened part we follow out the branches of the blood-vessels situated under the villous coat, in many individuals we see them diminished where they run under the softened part, and less patent and less filled with blood than in the healthy state. No thick mucous fluid is to be observed adhering to the softened parts. When the softening is perfect the colour of the villous membrane is pale, or white, frequently approaching to a bluish, apparently either continuous, or in long and narrow vibices, or rather in spots irregularly rounded, and lying more or less close to each other. These bluish portions appear prominent both to the sight and to the touch, and at their termination the healthy parts are seen well marked. The affected places of the mucous membrane are pale, very thin and soft, and converted into a kind of clear mucus, pale and semi-transparent. This mucous substance forms a thinner stratum than the proper membrane in a state of health; so that one examining it carelessly would suppose that the mucous coat had been destroyed and the cellular tunic left bare. If the product of the softening process has disappeared, the continuity of the villous membrane is found dissolved, with smooth margins, as if cut, and in its base the subjacent cellular tissue is perceived pale, and without injected blood-vessels. In some cases a large portion of the villous membrane has disappeared; then indeed the solution of continuity is not accurately circumscribed; for the membrane is more and more thinned at the edges, which seem irregular, and as if cut obliquely. The substance being lost in many places at the same time, what remains of the villous coat appears so softened, as not to have more consistence than the mucus commonly found in the intestinal canal. But when the softening is of less extent, no trace of it usually remains in the circumference of the dissolved part, the margins of which are smooth, and as it were accurately cut. The muscular coat and the peritoneum covering it are often more or less softened and pale; and the process of softening continuing, the intestinal tube is at length perforated.

"My friend, Dr. Allen Thomson has beautifully delineated, in a colored drawing made by him from the body of a child that died without having exhibited any symptoms of gastric irritation, in the Hospital des Enfans Trouves, a stomach which presented the lesion just described. In some portions of it where the destruction is not so complete as it is in the great sac, there is a very striking appearance, as if the fibres of the viscus were separated from each other by a grelatinous mucus. This seems to me to be the result of imbibition of the fluids from the cavity of the stomach, and was probably neither more nor less than the first inroad of the gastric juice upon the dead textures. This appearance has been much insisted on by Cruveilhier, as indicative of morbid action: but the farther consideration of this must be deferred to a subsequent part of this paper.

It would be improper to omit mentioning, that in several instances, as deliniated by Dr. Hope, and in most of the experiments of Dr. Carswell, there was a marked alteration in the color of the blood. "The blood vessels," says Dr. Carswell, "distributed on the softened parts, and also on every part where imbibition had occurred, no longer presented the red and blue ramifications, which are remarked in the healthy state, when they contain venous and arterial blood. They formed brownish, brownish-black, or almost pure black arborescences. In the great sac of the stomach, the brownish color was most common, while elsewhere, in proportion as an approach was made to the origin of these vessels, the color became deeper and deeper,—a circumstance evidently depending on the greater quantity of blood contained in the latter situations. In those parts, on the contrary, where the liquids could not come in contact with the vessels, or be conveyed to them by imbibition, the vessels preserved their natural color, and formed a very striking contrast by their redness with the deep tint of the others. The intercostal and diaphragmatic vessels where the great sac of the stomach rested on them, even though still covered by the peritoneum, presented the same coloration, which was confined exactly to the space covered by this portion of the stomach. The same state of the vessels was observed in the chest, in those places traversed by the liquids which had passed through the diaphragm. The form of the vessels was unaltered; and their parietes were even entire, where the coloration of the blood was complete. In this state the blood seemed less fluid than natural, but it had not separated into clots, or little striated stains, as is observed so frequently and so remarkably in man." In these facts every one must recognize the chemical action which it has been ascertained that all acids produce on the blood when mixed with that fluid.

It has generally been asserted that this lesion occurs most frequently in infants about the period of weaning, but the same appearances may be seen, with but trifling, if any variation, in adults of either sex. There are some slight differences in the accounts given by different authors of the symptoms of this so called disease, and, as nearly as I can understand them, the chief indicative signs, as they present themselves to view in children, may be enumerated as follows.

This disease is frequently preceded by premonitory symptoms, such as loss of appetite, peevishness, restlessness and want of sleep; the tongue is covered with a whitish or yellow fur, and a sour smell is perceived from the mouth, in the cavity of which aphthæ also frequently occur; constant diarrhœa comes on, in the progress of which the little patients become emaciated to a wonderful degree; the neck becomes wrinkled, and a dry and pituitous cough either has existed from the beginning or comes on at a later period. For the most part there is great thirst. The infants cry, or, their strength being much exhausted, lie on the back with the eyes fixed and half closed, their countenance being much disfigured, very pale and bloodless. The diarrhœa after a short time is often diminished, but soon returns with more violence; the skin of the hands and face becomes cold; the pulse irregular, small, scarcely to be counted; and the respiration quickened and short. The infants show no sign of pain upon gentle pressure of the abdomen, but at length die wasted away, their powers being exhausted, quietly and without any convulsion.

In other cases the disease begins with fever; the thirst is insatiable, the pulse much accelerated, with frequent diarrhœa; there is restlessness and frequent screaming, which in a short time passes into continued crying. The legs are drawn up upon the abdomen, which is hard and tumid, and on the application of external pressure, screams and distortions of the face sufficiently indicate pains in the abdomen; the emaciation increases; the countenance becomes pale, frequently also flushed, and wears an expression quite peculiar to suffering. The sleep, which at best is but light, is much broken by continual moaning and screams. The diarrhœa at last remits, convulsion and distortion of the eyes come on; the extremities, though sometimes hot, become at length cold; the pulse is frequent, irregular, small, and scarcely perceptible; occasionally somewhat hard. Cough and mucous rhonchus come on, the breathing becomes difficult, short and stertorous, and at length stupor and death conclude the scene. Other symptoms are frequently present, which indicate in many cases an inflammatory affection of the chest, and in others hydrocephalus."

"Besides the experiments on carniverous animals, and on the transference of the gastric juice to various organs formerly alluded to, the principal other experiments which I have seen Dr. Simpson perform, and in most of which I co-operated with him, are the following;—He procured the stomach of a pig, as being that of an omnivorous animal, and therefore most similar to that of man. The animal had been fed about an hour and a half previous to death, and the stomach was found to contain a considerable quantity of chyme. Portions of this matter, the acid character of which was indicated both by the change of color it effected on vegetable infusions, and by its smell, were introduced into different parts of the alimentary canal, such as the small intestine, cœcum, and colon, and these, along with the stomach itself, were kept in water about the temperature of 70 deg. Fahr. for between sixty and seventy hours. Upon examination after this lapse of time, there were no signs of putrefaction. It would indeed have been very singular had there been any, when we remember that the gastric juice is one of the most antiseptic fluids known, and the period of time since the death of the animal was not great. But although no putrefaction had occurred, we generally found the substance of the portions of great and small intestine much softened, somewhat darkened in color, and at particular places quite eroded, with numerous large perforations. The stomach itself was never found perforated, till it had been 90 or 100 hours under the action of the fluids contained in it,—a circumstance which may justly be attributed to the great thickness of the coats of this viscus in the sow. It was, however, generally observed to be much softened and discolored in its mucous coat some time before this period. This alteration of texture was sometimes limited by a distinct abrupt line which separated the natural from the dissolved parts. This seemed to occur in cases in which there was a considerable portion of air within the stomach, distending its cavity, and thus removing the superior parts of it from contact with the contained matters. In other instances where the gaseous fluids were allowed to escape, or were not generated in any quantity, the solution of the stomach was more extended, and at its edges presented the fringed and torn appearance so generally described. The stomachs of three dogs, which had been killed with large doses of hydrocyanic acid, about an hour or a little more after having been well fed, were subjected to a similar degree of warmth and moisture, and in the course of fifty hours were found to have undergone solution and perforation in many places.

Dr. Simpson also ascertained that this same phenomena of softening and perforation frequently takes place in the rennet, that is to say, in the stomach of the calf (which contains portions of curdled milk and gastric juice,) that has been well salted and dried in order to preserve it for coagulating milk. It is a matter of very frequent occurrence to find numerous large perforations in this substance, if it had not been kept in a perfectly dry condition.

Dr. Carswell having stated (Cyclopædia of Practical Medicine) that the introduction of an alkali into the stomach under experiment, and the consequent neutralization of the gastric acid prevents the erosion that would otherwise take place, we introduced into several portions of intestine the gastric liquor, mixed with a large quantity of magnesia, and found, contrary to what the statement of Dr. Carswell led us to anticipate, that as numerous and as large perforations occurred in these as in the other and adjoining portions of the same intestine submitted to a comparative trial, and containing gastric matter without the admixture of any alkali. In these cases there was also a very remarkable blackening of the intestinal texture, so as to give it the appearance of having been stained with China Ink, the cause of which I am unable to conjecture, not having been able to learn what communicates a black colour to magnesia. It is consonant, however, with the experience of nurses, that, on mixing magnesia with warm water, a yellow or grayish color is produced. Gruel is also found to become blackened on the addition of magnesia. It is right to mention, that in all our experiments, the portions of intestine were inverted and washed to clear them from any foreign substance they might contain, before the chymous matter from the stomach was introduced into them.

From these experiments, which were repeated by us on various occasions, I, for my own part, am fully convinced of the truth of an opinion I have for some time entertained, viz. that though we never find healthy gastric juice *not* containing acids, yet we must not thence infer that these are by any means the sole, or even the principal agents in effecting the solution of alimentary matters, or chymification.

In the few attempts Dr. Simpson and I made upon cats and rabbits to repeat the experiments of Dr. Camerer, by cutting the *par vagum* in the neck, we have as yet been unsuccessful in procuring his results. My own opinion is, that it is impossible ever to succeed in this; but as yet we have not a sufficient number of negative facts to overthrow the assertions of this author.

Having now given an outline of the opinions of the authors on the subject of this peculiar lesion, I beg to state the conclusion which I have formed from an attentive consideration of what has been written on it, as well as from the few original experiments I have detailed. I consider that the opinions of Mr. Hunter has been most amply proved to be correct by Dr. Carswell and others, including Dr. Gairdner and Mr. Cruveilheir themselves,—to wit, that the gastric juice is quite capable of producing, and frequently actually does produce, solution of the coats of the stomach after death, whatever designation we may give to this appearance, as pultaceous or gelatiniform ramollissement, mollestence of the stomach, gastromalacia, &c. This has been firmly established by numerous direct experiments on animals, whether carnivorous or herbivorous, and by the observation of a sufficient number of cases in man in which this state of the stomach has been found after sudden death from external violence while in the enjoyment of perfect health, and during the progress of natural digestion. The same cause, I think, has evidently operated in the other individuals in whom it occurred, who died from well-marked diseases of other organs; and we have the more reason for believing this, inasmuch as it has never been proved to have occurred where the stomach has been found empty at death, or where the examination of the body has been made a few hours merely after the termination of life; and all the authors who have taken notice of this circumstance, have particularly mentioned that the stomach contained some portion of alimentary matter or chyme. But we know that, independently of the presence of food, many irritating medicines will cause the secretion of the gastric juice; and every one knows that the readiest mode of procuring this fluid is by feeding an animal with pepper, or even with fragments of pebbles.

On the other hand, authors have failed entirely in establishing the existence of a peculiar morbid process causing perforation of the intestinal tube without ulceration; while yet no one would absolutely deny that there may exist some state of the viscus, rendering it a more easy prey to the action of the fluid it had itself formed. Again, we must remember that a more active condition of the gastric juice is by no means a proof of morbid action, for increase of power in a natural function in general only shows the higher perfection of the healthy state. Nor must we lose sight of the fact, that the peculiar train of symptoms, which we formerly enumerated, has been present in many individuals, in whom, after death, no such lesion of the stomach has been detected in any very appreciable degree.

Such, then, is the opinion I have endeavored to support,—one which is beginning to be the received opinion in Paris, and the same I have no doubt will soon be established as true. Thus M. Louis, in 1834, says in his "Examen de l'Examen de M. Broussais rélativement à la Phthisie et l'Affection Typhoide," (p. 15,) "I will not accept the congratulations which M. Broussais has addressed to me on the subject of ramollissement with thinning of the mucous membrane of the stomach, which I do not hesitate, says he, to explain by inflammation; for I have only said that this explanation seemed to me very admissible, without having considered it as being rigorously demonstrated. More lately, I have thought I could entertain new doubts relative to the justness of this interpretation. I have said, in fact, in my Recherches sur l'Affection Typhoide (i. 183,) that it seemed to me extremely probable, that, in a certain number of subjects, the lesion in question was not inflammatory, both because we do not find any evident traces of inflammation round the part which is softened, thinned, and pale; and because in these cases, the submucous cellular tissue participates in the alteration of the mucous membrane, and, though softened and thinned like it, is not inflamed in any part,—a circumstance which is quite contrary to what happens in the violent inflammation of the mucous membrane of the colon, for example, and that it is impossible to conceive according to the hypothesis that would make this lesion to be inflammatory. I shall add, that the memoir of Dr. Carswell is far from having strengthened me in the belief that the ramollissement which now occupies our consideration is the product of inflammation; that I ask for myself on the contrary, without being yet able to solve the problem rigorously, if the ramollissement of the stomach, and the perforation which is sometimes the consequence of it may not be in fact, as this honorable and talented physician has said, the product of a chemical action in the great majority of cases."*—*Edinburgh Med. and Surg. Journal.*

*To the kindness of Dr. William Thompson I have been chiefly indebted for the opportunities of consulting the different Theses I have had occasion to quote in the foregoing communication.

